# Identifying Gaps in Oral Care Knowledge, Attitudes, and Practices of Latinx Parents/Caregivers of Children With and Without Autism Spectrum Disorders

**DOI:** 10.1089/heq.2020.0078

**Published:** 2021-04-19

**Authors:** Lucía I. Floríndez, Dominique H. Como, Daniella C. Floríndez, Cheryl Vigen, Francesca M. Floríndez, Sharon A. Cermak

**Affiliations:** ^1^ USC Mrs. T.H. Chan Division of Occupational Science and Occupational Therapy in the Herman Ostrow School of Dentistry, University of Southern California, Los Angeles, California, USA.; ^2^ Willamette University, Salem, Oregon, USA.

**Keywords:** oral care, Latinxs, health equity, children, autism spectrum disorders

## Abstract

**Purpose:** This pilot study used data from a survey to examine the knowledge, attitudes, and practices about oral care of Latinx parents/caregivers of children with or without autism spectrum disorder (ASD) to identify gaps to focus future intervention.

**Methods:** Sixty English-speaking Latinx parents/caregivers who had a child between 4 and 14 years with or without ASD (*n*=31 ASD, *n*=29 typically developing [TD]) completed a questionnaire on oral health knowledge, practices, access to care, and demographics. Caregiver responses were compared, and gaps in knowledge and practices were identified.

**Results:** There were no significant differences in parent age, child age, income, insured status, or overall knowledge scores, only a significant difference in education (*p*=0.02), with the ASD group reporting less. Scores for knowledge, attitudes, access and practice were all nonsignificantly positively correlated, as was attitudes with access and practice. However, knowledge and attitudes were significantly negatively correlated. Additional significant findings were parents who had lower income and education, had lower oral knowledge scores, decreased frequency of dental visits, increased feelings of being discriminated against, children with increased fear of the dentist, and decreased ease of finding a dentist.

**Conclusion:** Factors such as income, education, ethnicity, and having a child with ASD can influence what Latinx parents and caregivers know about oral health and how their children experience receiving dental care. Latinx parents/caregivers of children with and without ASD report barriers to dental care, including difficulty attending visits or feeling stigmatized by their dental provider due to their ethnicity. Fear of the dentist is significantly correlated with ASD diagnosis and lower social demographics of the parent, and may contribute to a reduction in preventative oral care visits as well. Health care providers should consider these perspectives when providing care to this population to mitigate further oral health inequities.

## Introduction

Oral care is the most prevalent unmet health care need in children,^1^ and factors such as race/ethnicity, socio-economic status, and presence of a disability increase the risk for experiencing oral health disparities.^2–5^ Among individuals with special health care needs, those with autism spectrum disorder (ASD), which affects 1 in 54 children,^6^ comprise a population that is at risk for poor oral health.^7,8^ Parents have previously reported they have trouble finding a dentist for their children with ASD,^9^ as well as problems maintaining healthy oral care routines for their children due to their child's sensory sensitivities.^10^

For example, access to oral health care may be affected by the shortage of pediatric dentists who have training necessary to provide them with care,^11^ or who are willing to accept children with ASD as patients.^9,12,13^ Studies focusing specifically on oral care in Latinx children with ASD have been minimally addressed.^14^ However, there has been research on oral health issues in the Latinx^*^ population in general, including studies on TD populations. Research has shown that Latinxs have higher rates of dental caries, periodontal disease, and tooth loss than non-Latinx white individuals.^15^ Research also has shown that Latinxs are less likely to believe in the need for regular professional dental care, more likely to have misperceptions about oral health, and less likely to have access to care than the general population.^16–18^ In addition, Latinx children have the highest likelihood of never having seen a dentist.^19^

Findings from previous research with Latinx caregivers indicated that they attributed their children's caries to a variety of factors, including candy and sweets consumption, drinking sugary beverages, poor oral hygiene routines like limited tooth brushing, and extended bottle use.^20–22^ Other causes of oral health issues cited by the caregivers included poor nutrition, lack of dental insurance and high costs of dental care, lack of fluoride, genetics, and limited to no visits to the dentist for cleanings or routine care.^20–23^

Also, noteworthy is the information that the caregivers did *not* address. Misinformation and lack of knowledge among Latinx parents about the causes of caries and poor oral health in children continue to exist. Caregivers did not mention several factors contributing to tooth decay, including the role of bacteria and cariogenic content of foods on cavity development,^20^ or the significance of regularly practicing oral care behaviors.^22^ All studies acknowledged a need for increased parental knowledge about healthy oral care practices in the Latinx community as a means of cavity prevention in children.^20–22^

Other research has highlighted the importance of understanding the mechanisms underlying oral health behaviors that lead to caries in children,^24^ including exploring systemic barriers and stigmas related to ethnic identity or Latinx culture experienced by Latinx families.^14^ Indeed, understanding the oral health needs of Latinx children with and without ASD is a key area where more research is necessary, as both groups disproportionately experience oral health problems when compared to other ethnic groups and other groups of children with special health care needs.^3^

Gaps in Latinx parental oral care knowledge, attitudes, and practices may contribute to increasing oral health disparities and furthering health inequities experienced by Latinx children, warranting the need for more targeted education or culturally relevant intervention programs to address these areas. Therefore, the purpose of this study was to pilot a survey to examine the knowledge, attitudes, and practices of Latinx caregivers of children with and without ASD regarding oral health and oral care routines, to discuss how parents' knowledge and dental care utilization may impact the oral health and dental care experiences of their children, and to identify gaps to focus future intervention.

## Methods

The present data are part of a larger mixed-methods investigation designed to understand oral care practices and oral care health inequities among Latinx families living in Los Angeles. The first phase of the study involved qualitatively interviewing Latinx parents/caregivers of children with and without ASD to understand their experiences with oral care, including barriers to obtaining dental care or performing oral care routines; these data are published elsewhere.^14^ Following the qualitative phase, the quantitative portion of the study entailed developing a survey assessing oral care knowledge, attitudes, and behaviors to pilot with Latinx families; these findings are the focus of this article. Ethical approval was granted by the University of Southern California Institutional Review Board (HS-19-00170).

### Participants

All study participants were English-speaking parents/caregivers who self-identified as Latinx and had a child between 4 and 14 years old. Parents either had a child who was TD (no disability diagnoses or any significant medical issues) or a child with ASD based on parent confirmation of receiving a diagnosis of ASD from a licensed professional.

### Procedures

Parents/caregivers who agreed to participate completed a questionnaire about their knowledge, attitudes, and practices regarding oral care routines and oral health. To draw comparisons between groups, we surveyed parents/caregivers who had a child with ASD, as well as parents/caregivers with TD children to serve as the comparison group and gain a broader picture of knowledge, attitudes, and practices of Latinx families. Given the status of this study as a survey pilot, we did not calculate power; rather, we aimed to collect at least 50 questionnaires during the 4-week data collection period, lasting from March to April 2019.

The questionnaire, written in English, collected no identifying information, and was available in both a paper and electronic version. It had an explanatory cover letter for participants to read before completing it. Participants were recruited through word of mouth, partnership with local community centers, and via social media posts targeting support groups for parents. In instances where participants were approached in person, they were given the option to complete a paper form (which would be later input by a research team member) or input their answers directly into the electronic version. For electronic recruitment, a link to the survey was posted online for participants to elect to complete, along with a brief introductory post explaining the purpose of the survey. Participants were compensated with a $10 gift card for their time.

### Questionnaire development

Content for the questionnaire was informed by the literature and by data from interviews conducted by the first author with Latinx parents and caregivers with TD children and children with ASD.^14^ The results of these interviews were vital to providing culturally relevant content to include in the survey, namely questions about cultural norms like *familismo* and family inclusivity, pertinent food options, exploring feelings of mistrust of health care providers due to immigration status fear of deportation, culturally inspired remedies or practices, and language-related barriers to accessing oral care. In addition, as outlined by the Early Childhood Caries Collaborating Centers working group regarding essential content areas to include on oral health-related questionnaires, we included questions on “oral health knowledge, attitudes, oral health behavior, utilization, parent dental self-efficacy, quality of life, caregiver and family characteristics, and child characteristics”^25^ (p. 4).

We then consulted with experts in the field of pediatric dentistry, nutrition, Latinx health, cultural communication, occupational therapy, education, public health, biostatistics, and psychometrics to ensure thoroughness and accuracy in the content. Following face validity established by this expert review, we tested our questionnaire with six participants, who provided further feedback on refining questions before surveying the larger sample. Our final questionnaire consisted of 120 questions: 20 questions on demographics of the parent/guardian and characteristics of the child, 21 questions about oral health knowledge, 23 questions regarding oral care practices/behaviors of the parent and child, 8 questions regarding access to dental care/treatment, and 38 questions on dental attitudes and beliefs. All other questions collected descriptive content. This survey is available from the authors upon request for wider use.

Knowledge questions were formatted as True/False/I don't know, and scored as correct or incorrect, with “I don't know” answers marked as incorrect. Questions about attitudes and access used a Likert scale, where caregivers were asked to express their level of agreement with a statement, and the score was converted to a numerical value from 1 to 7 and then summed. Questions were reverse scored if they asked about negative attitudes, access beliefs, or practices.

### Analyses

The statistical significance of the differences in demographic information between the groups and correlations between summed knowledge, attitudes, access, or practice scores was tested using *t*-tests, Wilcoxon rank sum tests, and Fisher's exact tests as deemed appropriate. Between-group differences in knowledge, attitudes, and practices were analyzed using *t*-tests or Wilcoxon rank sum tests. Pearson Correlations were used to assess the relationship between demographic variables and oral care habits and beliefs. The analyses were performed using SAS statistical software version 9.4 (SAS Institute, Inc., Cary, NC). A two-sided 0.05 level of significance was utilized.

## Results

Table 1 provides descriptive characteristics of participating families. During the data collection window, we collected 60 total questionnaires for this pilot study. Seventeen questionnaires were completed in person; the remaining responses were submitted electronically. Participants were 60 parents/caregivers (31 with children with ASD and 29 TD children). All participants self-identified as Latinx. The majority of the responding parents/caregivers (90%) were female, with a mean age of 38.6 years (standard deviation [SD] 6.5 years), and most of the sample reported having dental insurance (73% of parents and 95% of children). The mean age of the 60 children was 8.5 years (SD ±3.2 years). There were no significant differences in age of parent, age of child, insured status, or level of income between the ASD and TD groups. There was a significant difference in level of education completed (*p*=0.02), where the ASD group reported less years of education completed than the TD group. However, each group had >80% attend at least some college. In the TD group, a higher percentage of parents had graduate degrees (38%) compared to the ASD group with 10% of parents having graduate degrees.

**Table 1.** Demographics of Total Sample and by Autism Spectrum Disorder and Typically Developing Groups

	Total	ASD	TD	*p*
*N*	60	31	29	
Child age^a^	8.5 (3.2)	8.6 (3.2)	8.4 (3.3)	0.75
Parent age^a^	38.6 (6.5)	38.0 (5.9)	39.2 (7.2)	0.48
Child overall health^b^				
Excellent	11 (18)	3 (10)	8 (28)	0.06
Very good	18 (30)	9 (29)	9 (31)	
Good	24 (40)	14 (45)	10 (34)	
Fair	6 (10)	4 (13)	2 (7)	
Poor	1 (2)	1 (3)	0	
Parent overall health^b^				
Excellent	1 (2)	1 (3)	0	0.75
Very good	20 (33)	10 (32)	10 (34)	
Good	21 (35)	12 (39)	9 (31)	
Fair	12 (20)	4 (13)	8 (28)	
Poor	6 (10)	4 (13)	2 (7)	
Parent dental insured status^c^				
Has insurance	44 (73)	22 (71)	22 (76)	0.77
No insurance	16 (27)	9 (29)	7 (24)	
Child dental insured status^c^				
Has insurance	57 (95)	29 (94)	28 (97)	1.0
No insurance 3 (5)		2 (6)	1 (3)	
Ancestry				
Argentina	2	1	1	
Chile	1	1	0	
Columbia	1	1	0	
El Salvador	6	1	5	
Guatemala	2	1	1	
Honduras	2	2	0	
Mexico	19	11	8	
Panama	1	0	1	
Paraguay	1	1	0	
Peru	1	0	1	
US	22	11	11	
Unknown	2	1	1	
Siblings^c^				
Yes	43 (72)	20 (65)	23 (79)	0.26
No	17 (28)	11 (35)	6 (21)	
Education^b^				
<HS	5 (8)	3 (10)	2 (7)	0.02^*^
Some HS	6 (10)	3 (10)	3 (10)	
Some college	20 (33)	15 (48)	5 (17)	
Bachelors	15 (25)	7 (23)	8 (28)	
Postgraduate	14 (23)	3 (10)	11 (38)	
Household income^b^				
<20,000	10 (17)	6 (19)	4 (14)	0.06
20,000–34,999	6 (10)	3 (10)	3 (10)	
35,000–49,999	8 (13)	6 (19)	2 (7)	
50,000–74,999	8 (13)	5 (16)	3 (10)	
75,000–99,999	7 (12)	5 (16)	2 (7)	
100,000+	21 (35)	6 (19)	15 (52)	

^a^ Mean (standard deviation); *t*-test.

^b^ *N* (%); Wilcoxon rank sum test.

^c^ *N* (%); Fisher's exact test.

^*^ *p*<0.05.

ASD, Autism Spectrum Disorder; TD, typically developing.

Table 2 provides information on the oral care habits of the families, and how they answered questions reflecting their attitudes toward oral care and knowledge about certain key topics. The data are presented for the whole sample, as there were no significant differences in the answers between the ASD and non-ASD groups. Overall, the majority of parents (78%) answered the question about milk bottle feeding correctly; however, only half of the sample (50%) correctly answered the question comparing fruit juice to sugary candy. Additionally, parents seemed to trust their child's dentist, but generally wished the dentist had more flexible appointment times available to them (for both questions, Median response was “Agree,” with an interquartile range of “Neutral” to “Strongly Agree”). Parents with children with ASD reported that their child's ASD diagnosis made oral care activities such as tooth brushing or flossing difficult (Median response was “Agree,” with an interquartile range of “Somewhat Agree” to “Strongly Agree”). Across both groups, parents also generally reported both their own oral health and that of their child as “Good.”

**Table 2.** Oral Care Practices and Attitudes in Both Autism Spectrum Disorder and Typically Developing Groups

Statement/question	*N* (%)	ASD	TD
Giving my child milk before bedtime to help them sleep does not impact their oral health.	47 (78)	25 (81)	22 (76)
Drinking fruit juice is better than eating candy for your teeth.	34 (57)	18 (58)	16 (55)
	*N* (% yes)		
Does your child have a regular dentist?	49 (82)	22 (71)	27 (93)
Median (IQR) 1=Strongly Disagree, 2=Disagree, 3=Somewhat Disagree, 4=Neutral, 5=Somewhat Agree, 6=Agree, 7=Strongly Agree			
Finding a dentist for my child is easy.	5 (3, 6)	3 (1, 6)	6 (4, 6)
I wish my child's dentist had more flexible appointment times that better match my schedule	6 (4, 7)	6 (4, 7)	6 (4, 6)
I trust my child's dentist.	6 (4.5, 7)	5 (4, 7)	6 (5, 7)
Dental care for my child is affordable.	5 (3, 6)	5 (3, 7)	5 (3, 6)
Getting dental insurance for my child is easier than getting dental insurance for myself.	5 (4, 6)	5 (4, 6)	4 (4, 6)
I prefer that my child's dentist is the same race/ethnicity as me.	4 (2, 4)	4 (2, 4)	4 (2, 4)
I think my family's race/ethnicity negatively influences how my child is treated at the dentist.	2 (1.5, 5)	2 (1, 5)	2 (2, 5)
My child's ASD diagnosis makes oral care activities like tooth brushing or flossing difficult. *(asked only of ASD parents)*	6.5 (5, 7)	7 (6, 7)	5 (4, 6)
Most children eventually develop dental cavities.	5 (3, 6)	5 (3, 6)	5 (3, 6)
I prioritize my child's dental health over my own dental health.	6 (5, 7)	6 (4, 7)	6 (5, 7)
Median (IQR) 1=Never, 2=Weekdays, 3=only Once daily, 4=Twice daily, 5=Three times daily, 6=More than three times per day			
How often does your child usually brush their teeth?	4 (3, 4)	3 (3, 4)	4 (4, 4)
How often do you usually brush your teeth?	4 (3, 4)	4 (3, 4)	4 (4, 4)
	Median (IQR)		
How many times have seen a dentist for a cleaning in the last 12 months?	1 (0, 2)	1 (0, 2)	1 (1, 2)
Median (IQR) 1=Poor, 2=Fair, 3=Good, 4=Very Good, 5=Excellent			
In general, would you say your oral health is:	3 (2, 4)	3 (2, 4)	3 (2, 4)
In general, would you say your child's oral health is:	3 (2, 3)	3 (2, 3)	2 (1, 3)

Table 3 provides results about overall knowledge, attitudes, practice, and access scores and their association with various demographic variables using *t*-tests. Although there was only one question which all respondents answered correctly (“Regular tooth-brushing helps prevent tooth and gum problems”), most participants had relatively good knowledge overall. The exception was for a question asking if dental problems were related to heart disease, stroke, diabetic complications, and lung cancer; most participants answered incorrectly and did not associate dental problems with other conditions.

**Table 3.** Relationship of Knowledge, Attitudes, Practice, and Access Scores with Demographic Information

	ASD	TD	*p*^a^	Sibling	No sibling	*p*	Education high^b^	Education low^b^	*p*	Income high^c^	Income low^c^	*p*
Knowledge	14.8 (4.4)	14.7 (4.7)	0.92	14.2 (4.6)	16.3 (3.7)	0.10	16.6 (2.9)	13.1 (5.0)	0.002^*^	16.6 (2.7)	13.2 (5.1)	0.002^*^
Attitudes	2.5 (0.2)	2.4 (0.2)	0.06	2.5 (0.2)	2.4 (0.2)	0.27	2.5 (0.2)	2.5 (0.2)	0.35	2.4 (0.2)	2.6 (0.2)	0.001^*^
Access	22.9 (4.5)	22.6 (3.4)	0.76	23.3 (4.1)	21.6 (3.5)	0.14	22.8 (3.7)	22.8 (4.2)	1.00	23.1 (3.3)	22.5 (4.5)	0.54
Practice	55.5 (7.2)	55.2 (5.6)	0.86	55.6 (5.9)	54.8 (7.6)	0.65	56.6 (6.0)	54.2 (6.6)	0.16	56.6 (5.6)	54.3 (6.9)	0.19

^a^ *t*-Test.

^b^ High = bachelor's degree − graduate degree/post graduate education; low = less than high school − some college/associate's degree.

^c^ High = $75,000+; low = <$75,000.

^*^ Indicates significant results.

Mean knowledge scores between ASD and TD groups were not significantly different, with a mean of 14.8 (SD 4.4) for the ASD group and a mean of 14.7 (SD 4.7) for the TD group. The breakdown of scores for each question of the knowledge portion is available from the authors. Parents who had less education (less than high school − some college/associate's degree) and lower family income (<$75,000) levels had significantly lower overall knowledge scores than participants with higher income (*p*=0.002) and more education (*p*=0.002). A score for attitude was derived from the mean of the items rather than the sum of the items. Using Pearson correlations, total scores knowledge, access, and practice were all nonsignificantly positively correlated with each other, as was attitudes with access and practice (*r'*s=0.07–0.21, *p'*s=0.11–0.59). However, knowledge and attitudes were significantly negatively correlated (*r*=−0.44, *p*=0.0004). This indicates that the higher the overall knowledge, the lower the attitudes toward oral health.

Table 4 presents the impact of demographic factors such as income level and ASD diagnosis on oral care attitudes and practices. We did not analyze insured status against attitudes or practice, as most children in our sample were insured (95%). Wilcoxon rank sum analysis showed that children from low education (*p*=0.001)/low income (*p*=0.0001) families were less likely to go to the dentist. More difficulty finding a dentist was significantly associated with ASD diagnosis (*p*=0.005) and lower parent education (*p*=0.04), while difficulty securing dental insurance was significantly associated with lower parent education (*p*=0.001) and lower income levels (*p*=0.024). Feeling unequal treatment at the dentist were significantly associated with lower income level of parent (*p*=0.0004). Notably, children with ASD (*p*=<0.0001) and children from low education (*p*=0.02)/income (*p*=0.0001) families were more likely to be fearful of going to the dentist than children without ASD or children from high education/income families.

**Table 4.** Impact of Demographic Factors on Oral Care Attitudes, Behaviors, and Practices

Statements about attitudes/beliefs/practices	ASD	TD	*p*^a^	Education high^b^	Education low^b^	*p*	Income high^c^	Income low^c^	*p*
1. How many times has your child seen a dentist for a cleaning in the last 12 months?	2 (1, 2)	2 (1, 2)	0.08	2 (2, 2)	1 (1, 2)	0.001^*^	2 (2, 2)	1 (1, 2)	0.001^*^
2. My child is fearful of going to the dentist	6 (4, 7)	2 (1, 5)	<0.0001^*^	2 (2, 5)	5 (2, 7)	0.02^*^	2 (1, 5)	5.5 (4, 7)	0.0001^*^
3. Finding a dentist for my child is easy	3 (1, 6)	6 (4, 6)	0.005^*^	6 (3, 6)	4 (2, 5)	0.04^*^	6 (3, 6)	4 (2, 6)	0.09
4. Getting dental insurance for my child is easier than getting dental insurance for myself.	4 (4, 6)	5 (4, 6)	0.18	4 (4, 5)	6 (4, 7)	0.001^*^	4 (4, 5)	6 (4, 6)	0.024^*^
5. I think my family's race/ethnicity negatively influences how my child is treated at the dentist.	2 (1, 5)	2 (2, 5)	0.71	2 (1, 4)	2 (2, 5)	0.36	2 (1, 2.5)	4 (2, 5.5)	0.0004^*^
6. My child's food preferences make eating nonsugary foods difficult	6 (5, 7)	4 (2, 5)	0.003^*^	4 (4, 6)	5 (4, 6)	0.18	4 (4, 6)	5 (4, 6)	0.18

Data shown as median (interquartile range).

1=Strongly Disagree, 2=Disagree, 3=Somewhat Disagree, 4=Neutral, 5=Somewhat Agree, 6=Agree, 7=Strongly Agree.

^a^ Wilcoxon rank sum test.

^b^ High=bachelor's degree − graduate degree/post graduate education; low=less than high school − some college/associate's degree.

^c^ High=$75,000+; low=<$75,000.

^*^ Indicates significant results.

**FIG. 1.**
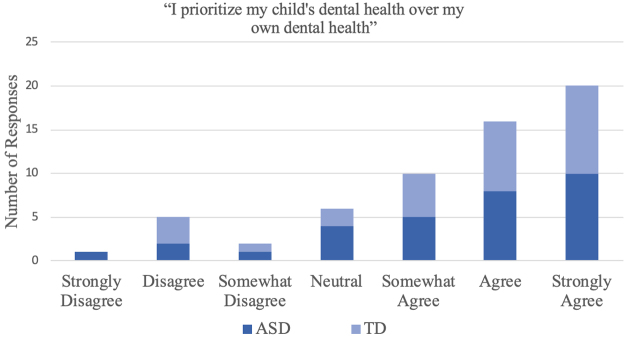
Frequency response to question, “I prioritize my child's dental health over my own dental health.”

When asked about prioritizing the oral health needs of their child over their own, reflecting the Latinx cultural value of *familismo*,^26^ a majority of parents (*n*=46 or 76.7% of the sample) indicated that they agreed with the statement. The frequency of responses to this question is presented in Figure 1.

## Discussion

The findings of this study highlight factors that affect dental care utilization in a sample of Latinx families, which include dental knowledge, family or individual behaviors, and culturally informed beliefs, attitudes, and practices, such as diet, feelings about health care providers, and concern for oral health. This research corroborates the impact that social determinants of health, such as education and income, have on persisting oral health disparities. However, it also offers deeper insights from a population underrepresented in current research, Latinx parents with children with and without a disability, on issues surrounding dental care, access, and perceptions of care provided.

First, the findings of this study confirm socioeconomic barriers, such as education, discrimination due to ethnic status, and income, impeding oral health care for Latinos. Economic resources, such as employment, reliable transportation, insurance, and income are essential for accessing and maintaining dental health,^27–30^ particularly in the United States, where employment is often directly tied to insured status. As most of the children in our sample were insured (95%), we were unable to determine whether insured status impacted attitudes or practice in our sample. However, previous research has shown that uninsured Latinx children had lower dental care utilization in a year than insured children,^28^ and established that lack of insurance and high cost of dental services were a major barrier for Latinx families,^31^ which were sentiments shared by our sample.

Understanding the importance of education levels and culturally related beliefs were also important findings of our study. Lower maternal education level has previously been correlated with negative oral health beliefs and practices in a Latinx sample,^18^ and the present findings also indicate that families with low education/income report having more difficulty finding dentists, receive dental cleanings less frequently, and feel more stigma and unequal treatment by their dental care provider due to identifying as Latinx than higher income families. This was true for Latinx families of both TD children and children with ASD. Overall knowledge scores negatively correlated with attitude scores. An interpretation may be that participants who knew more about their dental health recognized the barriers that they faced,^32^ as previous research has shown that Latinx parents can have fatalistic attitudes toward the oral health of their children^33^ due to perceived barriers to obtaining dental care. These fatalistic attitudes toward oral health are exacerbated by factors such as acculturation level, language, and cultural beliefs about not needing dental care, or mistrusting their health care provider.^34,35^ In these cases, creating socioculturally appropriate interventions, including those utilizing community health workers and participatory methods, have shown evidence at being effective in impacting underserved Latinx communities in need of oral care education programs.^36^

Another key finding of this study was describing which child patient was more likely to fear the dentist. Fears and phobias are commonly reported in ASD research,^37^ including at the dentist,^10^ and among Latinx patients with limited prior experience receiving dental care.^35,38^ Similarly, the present findings show that ASD diagnosis, low income, and low education are all associated with child's higher level of fear of the dentist. This fear of the dentist is likely to make dental visits more stressful, adding to the health disparities experienced by families and child patients who already suffer from multiple inequalities, and perpetuating the cycle of oral health disparities.

The last significant finding of this work is the majority of the sample (76%) reporting that they put the dental needs of their children first. This finding held true across all education levels, income levels, country of origin, and regardless of ASD diagnosis, suggesting that this *sacrifice* made by parents for promoting the health of their children that is broadly shared within Latinx families. While observed in Latinx parents in other care settings,^39^ more testing would have to be done to draw conclusions as to whether this finding is exclusive to Latinx parents, this finding has larger implications about the impact of *familismo*, the Latinx cultural value of placing family first,^26^ and how the Latinx parents surveyed supported this concept.

Strengths of this work are the inclusion of a broad range of health factors (i.e., knowledge, attitudes, practices, access, and culture) on oral health outcomes in a Latinx population. This survey also represents a critical integration of culturally based concepts into a questionnaire. Limitations of the study include a small sample size and lack of Spanish translation, indicating that these results are limited to English-speaking Latinx families with children with and without a disability, and are not further generalizable. Given that there are over 10 million Spanish-speaking households in California, by excluding Spanish speakers from our study, we are omitting a considerable data source, and are unable to comment further on how language barriers may be a critical factor in understanding disparities related to perceived discrimination, immigration status, and access to health care. In addition, we collected no identifying information from respondents. Thus, we have no way to ensure that parents only completed the survey once, medically confirm the ASD or TD diagnosis in each child, or further explore issues related to undocumented status of our sample. Future research should consider translating the questionnaire and expanding the sample size, as well adding a component to confirm ASD diagnosis.

## Conclusion

Latinx parents/caregivers of children with and without ASD report barriers to dental care that perpetuate oral health disparities, including difficulty accessing dental care or feeling stigmatized by their dental provider due to their ethnic identity. Fear of the dentist is significantly correlated with ASD diagnosis and lower social demographics of the parent, including income and level of education completed, and may contribute to a reduction in preventative oral care visits as well. Thus, accurate assessment of Latinx caregivers' understanding about children's oral health is important to guide in the planning and implementation of future culturally tailored educational and behavioral interventions to address oral health disparities in this underserved and underresearched population.

## Abbreviations Used

ASD: autism spectrum disorder
TD: typically developing

## Notes

^*^ Throughout this article, we use *Latinx*, a gender neutral term to indicate someone of Latino/a ethnicity.
